# Removal of Ibuprofen from Contaminated Water by Bioaugmentation with Novel Bacterial Strains Isolated from Sewage Sludge

**DOI:** 10.3390/microorganisms13081927

**Published:** 2025-08-18

**Authors:** Inés Aguilar-Romero, Alba Lara-Moreno, Fernando Madrid, Jaime Villaverde, Esteban Alonso, Juan Luis Santos, Esmeralda Morillo

**Affiliations:** 1 Institute of Natural Resources and Agrobiology of Seville, Spanish National Research Council (IRNAS-CSIC), 41012 Seville, Spain; ines.aguilar@csic.es (I.A.-R.); fmadrid@irnase.csic.es (F.M.); jvillaverde@irnase.csic.es (J.V.); 2 Department of Microbiology and Parasitology, Faculty of Pharmacy, University of Seville, 41012 Seville, Spain; alara9@us.es; 3 Department of Analytical Chemistry, University of Seville, C/Virgen de África 7, 41011 Seville, Spain; ealonso@us.es (E.A.); jlsantos@us.es (J.L.S.)

**Keywords:** ibuprofen biodegradation, wastewater, bacterial remediation, metabolites

## Abstract

Ibuprofen (IBP), one of the most consumed drugs in the world, is only partially removed in Wastewater Treatment Plants (WWTPs). Its presence in effluents and sewage sludge introduces IBP into the environment. It is imperative to continue research on IBP degraders that can be used in the future to eliminate IBP at the WWTP level. This study describes the use of nine specific IBP-degrading bacteria isolated from sewage sludge (*Achromobacter denitrificans*, *Bordetella petrii*, *Brucella tritici*, *Curtobacterium flaccumfaciens*, *Microbacterium paraoxydans*, *Pseudomonas citronellolis*, *Pseudomonas nitroreducens*, *Shinella zoogloeoides*, *Stenotrophomonas acidaminiphila*) for the removal of IBP from water. Their half-maximal inhibitory concentration of IBP for bacterial growth (IC_50_) revealed a high level of IBP tolerance. Degradation of IBP (10 mg L^−1^) was effective for all the strains using glucose as a secondary substrate. Seven of the nine strains were shown to be IBP degraders for the first time by our research group, highlighting *A. denitrificans* CSW15, with almost 47% IBP degraded, and *C. flacumfaciens* CSW18, with 32.2% after 28 days. Three IBP transformation products were identified: 1-hydroxyibuprofen (1-OH-IBP), 2-hydroxyibuprofen (2-OH-IBP), and carboxyibuprofen (CBX-IBP). A comparison of the effectiveness of IBP degradation by the nine isolates with most other IBP-degrading bacteria previously reported was carried out.

## 1. Introduction

Ibuprofen (IBP), a widely used non-steroidal anti-inflammatory drug (NSAID), is one of the most consumed pharmaceuticals worldwide for the treatment of pain and inflammation. After its use by humans, it is excreted and enters wastewater treatment plants (WWTPs), where, despite the application of various treatment technologies, complete removal of IBP is not achieved, and a significant amount is released into the environment [1]. Through the continuous discharge of treated effluents from WWTPs, IBP has been detected in rivers, lakes, and even groundwater, affecting water quality and the organisms that depend on it. Moreover, IBP is relatively stable and does not easily break down in the environment, which contributes to its persistence and classification as an emerging contaminant [2]. For this reason, the European Union has identified IBP as a candidate for inclusion in the priority substance list under the Water Framework Directive [3], as recognized by the Priority Substance Directive [4] and the European Commission [5]. IBP can also be found in agricultural soils due to the use of treated wastewater for irrigation, as well as the application of sewage sludge from WWTPs used as fertilizer [6], with potential implications for food chain transfer.

Given the widespread use and ecological risks associated with IBP, its removal during wastewater and sludge treatment is of critical importance, since WWTPs are the primary entry point for IBP into the environment, with the advantage that WWTPs offer more controlled conditions (pH, temperature, carbon sources, etc.) than natural environments. Various physical and chemical methods have been proposed for IBP removal, mainly for water and wastewater, but the most promising approaches are biological methods, which offer a cost-effective and environmentally sustainable alternative, particularly those employing specific microbial strains or consortia isolated from environments with long exposure to IBP, such as WWTPs [7]. However, only a limited number of IBP-degrading isolated bacterial strains have been reported and the majority of them were identified only a few years ago [8,9,10,11,12,13,14,15,16,17].

Although most degradation studies have been conducted under laboratory conditions and have not been applied in real environmental settings, it is essential to continue researching new bacteria that specifically degrade IBP to scale up these bioremediation technologies in WWTPs in the near future under the specific environmental conditions required for their microbial activity. This study aims to investigate, in depth, the ability of nine pure bacterial strains, previously isolated from sewage sludge from a WWTP by our research group [18], to degrade IBP in contaminated water. Before degradation experiments, the tolerance of each strain to IBP will be quantified through IC_50_ determination. Subsequently, IBP biotransformation kinetics will be evaluated and the possible transformation products formed during the process will be identified. A comparison of their degradation effectiveness with the rest of IBP-degrading bacteria in the literature will be carried out, as well as a comparative phylogenetic analysis of these novel strains based on 16S rRNA gene sequences to search for taxonomic similarities with other IBP-degrading bacteria.

## 2. Materials and Methods

### 2.1. Chemicals and Bacterial Strains

Analytical standards of IBP (C_13_H_18_O_2_, purity > 98%, CAS: 15687–27–1) and IBP sodium salt (C_13_H_17_O_2_Na, purity > 98%, CAS: 31121–93–4) were purchased from Sigma-Aldrich (Madrid, Spain). The Luria–Bertani (LB) broth and the mineral salt medium (MSM) were prepared with analytical-grade chemicals (Sigma-Aldrich, Madrid, Spain) and autoclaved at 121 °C for 20 min before use. LB broth contained (per liter of deionized water): 10 g tryptone, 5 g yeast extract, and 10 g NaCl (BD Difco™, Fisher Scientific, Pittsburgh, PA, USA). MSM was composed of the following components (per liter of deionized water): 0.5 g of KH_2_PO_4_, 0.5 g of K_2_HPO_4_, 0.01 g of NaCl, 0.2 g of MgCl_2_⋅6 H_2_O, 0.02 g of CaCl_2_, 1 g of (NH_4_)_2_SO_4_, 0.339 mg of MnSO_4_, 0.428 mg of ZnSO_4_, 0.347 mg of (NH_4_)_6_Mo_7_O_24_⋅4 H_2_O, 0.4 mg of CoCl_2_⋅6 H_2_O, 5 mg of FeSO_4_⋅7 H_2_O, 0.2 mg of CuSO_4_⋅5 H_2_O, and 10 mg of EDTA. All solvents used were HPLC-grade.

IBP-degrader bacterial strains used were isolated and identified by Aguilar-Romero et al. [18] from an IBP-degrader consortium C7 obtained from fresh sewage sludge collected from a WWTP in Seville (Southwest Spain), described previously by Vargas-Ordóñez et al. [19]. The nine bacterial strains under study were isolated as described by Aguilar-Romero et al. [18], with details about cultivation and isolation conditions, pure cultures acquisition, stock culture preservation and phylogenetic analysis.

These nine bacterial strains were deposited in the GenBank database with the following accession numbers: *B. tritici* CSW06 (OQ859973), *B. petrii* CSW07 (OQ859974), *M. paraoxydans* CSW08 (OQ859975), *P. citronellolis* CSW09 (OQ859976), *S. acidaminiphila* CSW10 (OQ859977), *S. zoogloeoides* CSW12 (OQ987996), *P. nitroreducens* CSW13 (OQ987997), *A. denitrificans* CSW15 (OQ987999), *C. flaccumfaciens* CSW18 (OQ988002). They were selected to evaluate the biotransformation of IBP since they all demonstrated previously biodegradative abilities [18].

Phylogenetic tree analyses based on the 16S rRNA sequences were performed using the maximum-likelihood and neighbour-joining algorithms with 1000 bootstrap replications in MEGA version 11 software [20] to show the evolutionary distances among the different bacterial isolates (based on best BLAST matches).

### 2.2. Inoculum Preparation

Bacterial strains of the isolates were cultivated in LB medium in the presence of IBP sodium salt (200 mg L^−1^) (30 ± 1 °C, 150 rpm) and harvested at the beginning of the stationary phase by centrifugation at 7000 rpm (10 min). The pellets obtained were washed with MSM to completely remove the IBP and LB previously added, and then resuspended in MSM.

Bacterial growth was monitored by the optical density (OD) at 600 nm at the beginning of the assay using a VWR UV-3100 spectrophotometer (Avantor, Radnor, PA, USA), and by colony-forming units (CFU) from serial dilutions on LB medium plates. The initial cell density of each strain added to the degradation experiments was 10^8^ CFU mL^−1^.

### 2.3. Inhibitory Concentration of IBP for Bacterial Growth

The half-maximal inhibitory concentration (IC_50_) for bacterial growth refers to the concentration of IBP that reduces bacterial growth by 50%. This value was determined using duplicate batch cultures in sterile 100 mL Erlenmeyer flasks. Each flask contained 20 mL of LB medium, IBP sodium salt at varying concentrations (10, 100, 500, 1000, 3000, and 5000 mg L^−1^), and an initial bacterial inoculum with an OD_600nm_ of 0.1. In addition, a duplicate culture without IBP was included as a reference for normal bacterial growth.

The assay was incubated under aerobic conditions with orbital shaking at 180 rpm and 30 °C for 24 h. Bacterial growth in each culture was measured by optical density (OD_600nm_) using a VWR UV-3100 spectrophotometer after the incubation period. Based on these measurements, bacterial viability (%) was calculated as the OD_600nm_ of the culture in the presence of drug concentration divided by the OD_600nm_ of the culture without IBP. IC_50_ values were estimated by plotting the logarithm of IBP concentration (log [IBP]) (mg L^−1^) against bacterial viability (%) using Microsoft Excel.

### 2.4. Biodegradation Assays in Solution with Isolated IBP-Degrading Bacteria

Isolated bacterial strains were inoculated in flasks with 20 mL of MSM spiked with IBP (10 mg L^−1^) at an initial cell density of 10^8^ CFU mL^−1^. Microcosms were incubated in triplicate on a temperature-controlled rotary shaker at 30 °C and 180 rpm under aerobic conditions for 28 days.

Taking into account that none of the nine bacterial strains under study were able to degrade IBP as a monosubstrate, they were inoculated in the presence of glucose (3 g L^−1^). Non-inoculated sterile controls were also prepared to observe any IBP abiotic degradation. For abiotic controls, solutions containing MSM were sterilized by autoclaving at 121 °C for 40 min, and sodium azide was added at an initial concentration of 200 mg L^−1^ to avoid biological activity.

At periodic intervals, the corresponding flasks were removed from the incubator to determine the remaining IBP concentration in solution. To break cell walls and recover IBP accumulated in the microbial biomass, flasks were frozen and thawed three times. Samples were centrifuged at 11,000 rpm for 2 min, and IBP concentration was determined in the supernatants as explained in Section 2.5.

### 2.5. Analytical Quantification of Ibuprofen and Its Transformation Products

IBP concentration was determined in the supernatant solutions as described in [17]. Briefly, a HPLC analyser with UV–Vis detector (LC-2010AHT, Shimadzu, Kyoto, Japan) was used, with a C-18 column (4 × 150 mm), mobile phase methanol: water (80:20) at pH 3 with orthophosphoric acid (1%), flow rate of 1.2 mL min^−1^, detection wavelength 210 nm and injection volume 25 µL. The IBP detection limit was 0.05 mg L^−1^.

The analysis of IBP metabolites was conducted using high-performance liquid chromatography coupled with tandem mass spectrometry (HPLC-MS/MS). Chromatographic separation was performed on an Agilent 1290 Infinity II HPLC system (Agilent, Santa Clara, CA, USA), equipped with a vacuum degasser, binary pump, autosampler, and a thermostated column compartment. The analytical column used was a Halo C18 (50 mm × 4.6 mm i.d., 2.7 µm particle size) from Teknokroma (Barcelona, Spain), protected by a matching Halo C18 guard column (5 mm × 4.6 mm i.d., 2.7 µm particle size).

The mobile phase consisted of water with 0.1% formic acid (solvent A) and acetonitrile with 0.1% formic acid (solvent B), operated under a gradient elution program as follows: 0–2 min, isocratic at 10% B; 2–4 min, linear gradient from 10% to 30% B; 4–8 min, linear gradient from 30% to 100% B; held at 100% B for 7 min; and re-equilibrated to initial conditions over 5 min. The flow rate was set at 0.6 mL min^−1^, with an injection volume of 10 µL, and the column temperature was maintained at 35 °C.

Mass spectrometric detection was carried out using an Agilent 6495 triple quadrupole mass spectrometer equipped with an electrospray ionization (ESI) source. The ionization parameters were as follows: capillary voltage 4000 V, drying gas flow rate 11 L min^−1^, drying gas temperature 350 °C, nebulizer pressure 40 psi, and fragmentor voltage 166 V. The instrument operated in both positive and negative ESI modes.

Two multiple reaction monitoring (MRM) transitions were selected for each target compound to enable both quantification and confirmation. The selected transitions and their corresponding quantifier/qualifier ion ratios are presented in Table S1. Optimized collision energies for each MRM transition are also provided in Table S1. The limits of quantification (LOQs) were 1 µg L^−1^ for 1-hydroxyibuprofen and 2-hydroxyibuprofen, and 10 µg L^−1^ for carboxyibuprofen.

### 2.6. Models of Biodegradation Kinetics

All IBP biodegradation curves were analyzed and fitted to the most appropriate kinetic models using the Excel tool developed by the FOCUS [21] workgroup for degradation kinetics. The fitting process employed the GRG non-linear algorithm of the Solver add-in (Microsoft), and parameter optimization was carried out using the least-squares approach.

Overall, the degradation patterns aligned well with two first-order kinetic models: the Simple First-Order (SFO) model and the biphasic First-Order model known as the Hockey-Stick (HS) model, described by the following equations:

Mt = M_0_ e^−kt^                                        (SFO)

DT_50_ = ln2/K                                        (SFO)

Mt = M_0_ e^−k1^ tb e^−k2(t-tb)^                         (HS)

DT_50_ = (ln 100/100 − 50)/K_1_                    if DT_50_ ≤ tb         (HS)

DT_50_ = tb + (ln 100/100 − 50) – K_1_ tb)/K_2_      if DT_50_ > tb      (HS)

In these models, Mₜ represents the IBP concentration (mg L^−1^) at a given time t, while M_0_ denotes the initial concentration immediately after spiking. The degradation rate constant is expressed as K (day^−1^). For the Hockey-Stick (HS) model, K_1_ and K_2_ correspond to the rate constants (day^−1^) associated with the rapid and slower degradation phases, respectively, and tb indicates the breakpoint time at which the rate changes. The DT_50_ value, or half-life, signifies the time required for the IBP concentration to decrease by 50% from its initial level.

### 2.7. Statistical Analysis

Standard deviations were calculated using Microsoft Excel for parameters such as biodegradation, bacterial viability and transformation products, and were included in the Figures for data comparison. The kinetic model applied in biodegradation assays was implemented using the Solver tool software from Microsoft’s statistical package. The suitability of this kinetic model was evaluated using scaled residual errors and Chi-square (χ^2^) tests, with a significance level set at *p* < 0.05.

## 3. Results and Discussion

### 3.1. Ibuprofen-Induced Inhibition of Bacterial Growth

**Figure 1:**
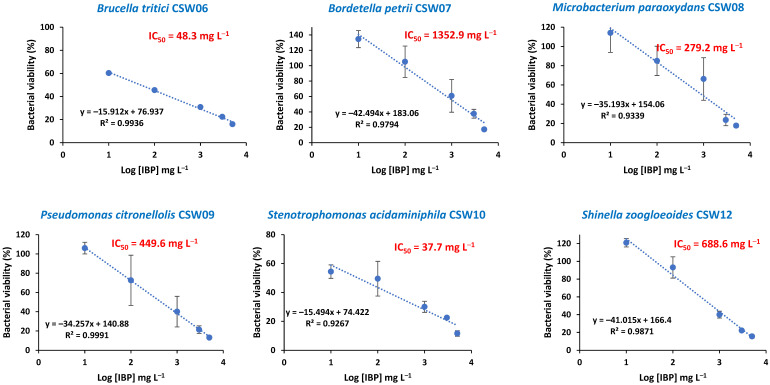

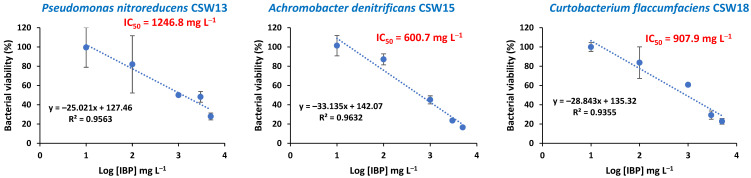
Graphical representation of the best-fitting linear curves for bacterial viability as a function of the logarithm of IBP concentration (log [IBP]) for the nine bacterial isolates. Error bars indicate standard deviation from three replicates.

IBP is widely recognized for its anti-inflammatory properties; however, several studies have also demonstrated that it can exhibit antibacterial activity [22]. This potential effect has been supported by research showing its ability to inhibit bacterial growth, as evidenced by the calculation of IC_50_ values against various bacterial strains. In this study, the IC_50_ was determined to assess the effect of different IBP concentrations (10, 100, 1000, 3000, and 5000 mg L^−1^) on the bacterial strains under study. The assay involved comparing OD_600_ values after 24 h of incubation in media with and without IBP. A linear correlation was observed between bacterial viability and the logarithm of IBP concentration across all strains (Figure 1).

Overall, the results indicate a high level of IBP tolerance among the isolates. The highest IC_50_ values were observed for *B. petrii* CSW07, *P. nitroreducens* CSW13, and *C. flaccumfaciens* CSW18, with values of 1353, 1247, and 908 mg L^−1^, respectively (Table 1). These were followed by *S. zoogloeoides* CSW12, *A. denitrificans* CSW15, *P. citronellolis* CSW09, and *M. paraoxydans* CSW08, with IC_50_ values of 689, 601, 450, and 279 mg L^−1^, respectively (Table 1).

These results suggest that these strains can tolerate high concentrations of IBP under nutrient-rich conditions and may, therefore, have strong potential for its biodegradation, taking into account that the maximum IBP concentration in influent and effluent municipal WWTPs observed by Wu et al. [7] in 25 countries were 39.8 mg L^−1^ and 0.059 mg L^−1^, respectively. Previous studies support this potential. Show et al. [23] and Wittich et al. [24] demonstrated the capacity of *P. citronellolis* and *M. paraoxydans* to efficiently degrade IBP, consistent with their high IC_50_ values observed in this study.

In contrast, comparative data are lacking for the other species tested. Notably, only two strains exhibited significantly low IC_50_ values: *Stenotrophomonas acidaminiphila* CSW10 and *Brucella tritici* CSW06, with values of 38 and 48 mg L^−1^, respectively (Table 1). These results suggest that these bacteria may be less effective in degrading IBP at high concentrations since differences in IC_50_ values reflect genetic and physiological variability that is key for the selection of strains in bioremediation processes [25].

Nonetheless, all strains were selected for further experimentation, as the IBP concentration to be tested in subsequent assays will be lower (10 mg L^−1^). As far as we know, no previous studies have reported IC_50_ values for the bacterial genera analyzed in this study in the presence of IBP, which highlights the novelty of our findings regarding their tolerance to IBP.

IBP has also been demonstrated to inhibit the growth of certain bacteria in laboratory settings, suggesting a potential antibacterial effect, particularly against specific strains. Some bacteria, like *Staphylococcus aureus* and *Paracoccus yeei*, have shown susceptibility to IBP at lower concentrations, while others, like certain *Enterobacter* strains, have exhibited resistance [26]. Marchlewicz et al. [10] showed that in the presence of 809 mg L^−1^ of this drug, there is a 50% inhibition of the growth of the *Bacillus thuringiensis* B1(2015b) strain, showing an IC_50_ value quite similar (or even lower) than those obtained for some of the nine bacteria under study (Table 1).

### 3.2. IBP Biodegradation in Aqueous Solution by Bacteria Isolated from Sewage Sludge

From the nine selected bacterial strains under study, none of them was capable of degrading IBP 10 mg L^−1^ when provided as the sole carbon source [18]. Anthropogenic pollutants tend to be resistant to biological degradation, and co-metabolic conditions are frequently applied [27], since the presence of a secondary substrate increases the biomass of the degrader microorganism and/or induces the presence of enzymes required for the degradation of the target substrate [28]. Other IBP-degrader bacteria have used glucose ([10,29,30]) or other substrates such as yeast extract ([11,23,31,32,33]), dextrose and peptone ([12,34]) or n-hexane [35] to stimulate microbial growth and enzyme production.

**Figure 2:**
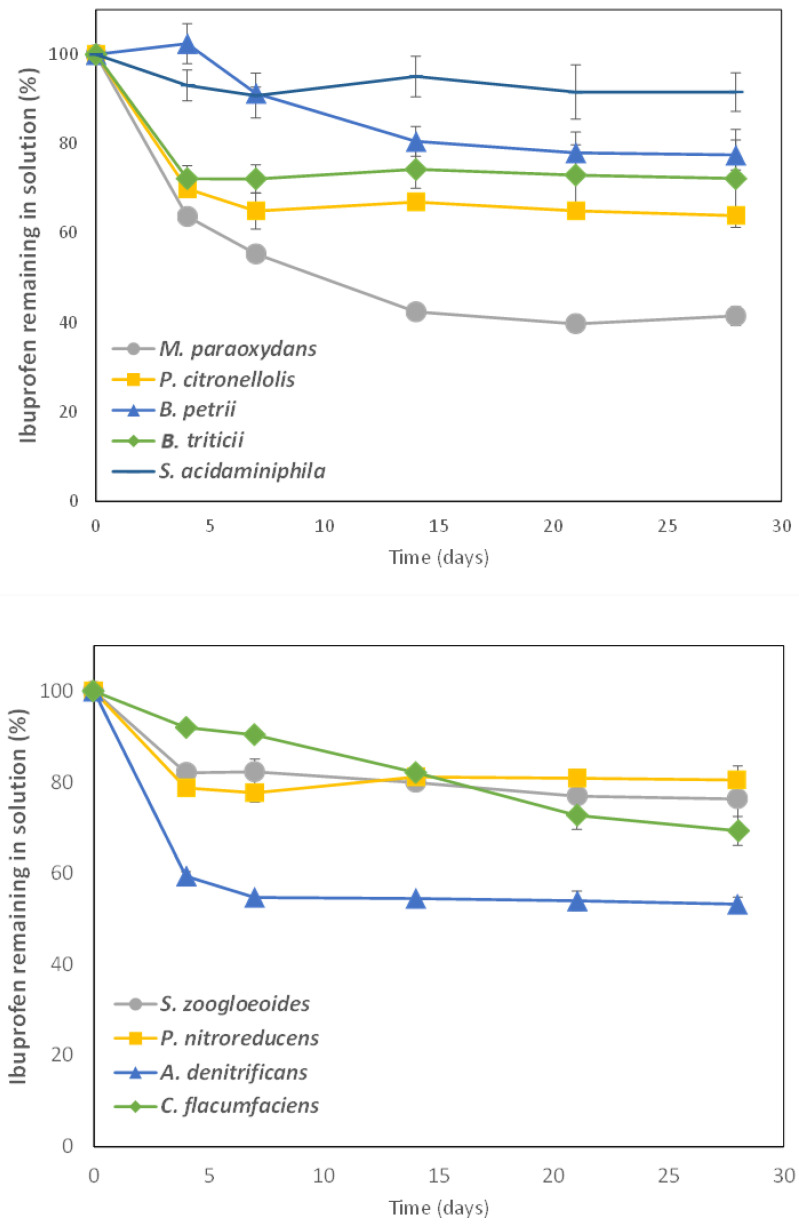
Ibuprofen (10 mg L^−1^) degradation capability by several bacterial strains isolated from consortium C7 in the presence of glucose (3 g L^−1^). Error bars indicate standard deviation from three replicates.

Taking into account that these nine bacterial strains were not able to degrade IBP as a monosubstrate, we selected glucose as a second substrate to be used as C and energy source. Complete IBP biodegradation curves using the different isolated bacterial strains were carried out during 28 days. The presence of 3 g L^−1^ glucose promoted IBP degradation for all these bacterial strains. The degradation curves obtained are shown in Figure 2 and the corresponding kinetic parameters are given in Table 2.

All the strains tested were able to degrade IBP in the presence of glucose but with a wide variation in the extent of degradation in 28 days (from 9% to 64%). Only two of the isolated bacteria, *M. paraoxidans* and *P. citronellolis*, had been previously reported to degrade IBP. *M. paraoxidans* CSW08 showed the best results, reaching almost 64% IBP degradation in 28 days and with a DT_50_ of only 12.5 days (Table 2). As previously mentioned, only recently did Show et al. [23] identify *M. paraoxidans* as an IBP degrader for the first time. There are not many references about *M. paraoxidans* as a degrader of organic contaminants [36], but there is more information about its role in removing heavy metals from different matrices [37].

*P. citronellolis* has also been very recently indicated as an IBP degrader [24], but only when the three isolated bacterial strains *P. citronellolis* RW422, 423 and 424 were added as a consortium, since none of the isolates were able to grow with IBP as the sole carbon and energy source. In the case of *P. citronellolis* CSW09, more than 36% IBP (10 mg L^−1^) was degraded in 28 days (Table 2). Another pharmaceutical compound, such as the synthetic estrogen 17α-ethynylestradiol has recently been demonstrated to be degraded by *P. citronellolis* SJTE-3 [38].

Our research group demonstrated for the first time that the rest of the isolated bacterial strains under study showed the ability to degrade IBP [18]. Among them, it is worth noting IBP degradation by *A. denitrificans* CSW15, with almost 47% degraded in 28 days and a DT_50_ value of 44.7 days (Table 2). This is the first time that *A. denitrificans* has been described as an IBP degrader, but it was demonstrated to increase the degradation of sulfamethoxazole and other sulphonamides [39,40], oxytetracycline [41], and other organic contaminants such as phenanthrene [42] or microplastics [43].

*C. flaccumfaciens* CSW18 was able to degrade up to 32.2% IBP but it had not been described previously as an IBP degrader. However, this bacterium is able to degrade various organic compounds, such as phenol (700 mg L^−1^ in 96 h) [44]. *C. flaccumfaciens* is a genus with a wide distribution, particularly in soils, where it is able to degrade structural polysaccharides, playing a role in the decomposition of organic matter in litter communities [45].

*B. petrii* is also described as an IBP degrader for the first time in this study, with CSW06 being able to degrade up to 24.5% IBP in 28 days, with a DT_50_ value of 61.2 days (Table 2). However, *B. petrii* is known for its ability to degrade a variety of organic compounds, such as aromatic hydrocarbons [46], or pesticides such as endosulfan [47] and 2,4-D [48]. *B. petrii* is also known to produce biosurfactants, which enhance the bioavailability of hydrophobic pollutants and help in their biodegradation [49].

*S. zoogloeoides* is a bacterial strain frequently isolated from sewage and activated sludge from WWTPs with the capacity to degrade organic contaminants such as pyridine [50], anthracene [51], or pharmaceuticals such as the estrogenic compound 17α-ethinylestradiol [52], but this is the first time that its ability to degrade IBP is shown (24% IBP degraded).

*P. acidaminiphila* is a bacterial strain frequently found in contaminated soils and also in activated sludge. Its degradative abilities have been demonstrated for a wide variety of pollutants, such as the herbicide glyphosate [53], the plasticizer dibutyl-phthalate (DBP) [54] or the antibiotic nitrofurantoin [55]. However, this is the first time that *P. acidaminiphila* CSW10 is reported as an IBP degrader, although with a very low degradation capacity (<10% in 28 days).

*P. nitroreducens* CSW13 is also reported for the first time as an IBP degrader, reaching almost 20% degradation (Table 2), although other *P. nitroreducens* strains have previously been reported as degraders of the insecticide chlorpyrifos [56] or the antidepressant fluoxetine [57].

The same applies to the bacterial strain *B. triticii,* but it is worth noting that this is a very poorly known species with few reported degradative abilities. Only recently has it been observed to degrade low-density polyethylene (LDPE) plastic [58], but *B. triticii* CSW06 was capable of degrading for the first time more than 27% IBP in solution in the presence of glucose (Table 2).

It should be noted in Figure 2 that those degradation curves which fit a biphasic first-order sequential model (Hockey-Stick, HS) show a value of K_2_ (the rate constant for the degradation of the slow fraction) that is extremely low (Table 2), indicating that from the value of tb (time at which the rate constant changes) IBP degradation is quite difficult, as in the cases of CSW09, CSW10, CSW12 or CSW15, and even null for some strains, such as *B. triticii* CSW06 or *P. nitroreducens* CSW13. This could probably be due to the toxic effects of IBP-generated biotransformation products and metabolites during the 28-day degradation process on these strains, affecting the kinetics of the degradation process, although this was not directly tested in this study.

Although the Inhibitory Concentrations of IBP for bacterial growth (IC_50_) values previously observed indicated a high tolerance level to increasing concentrations of IBP, metabolites were likely not present. The study of biotransformation products and/or metabolites generated will be addressed in a later section.

Table 3 provides a comparative study of the results obtained in the present study regarding the concentrations of IBP degraded by the nine bacterial strains with the results obtained by other researchers using different IBP-degrader bacteria. There are some strains whose degrading efficiency is much higher, such as those with proven ability to mineralize IBP: *Sphingomonas* sp. Ibu-2 (500 mg L^−1^ in 3d, [8,9]) or *Sphingopyxis granuli* RW412 (456.5 mg L^−1^ in 3d, [16]). Some others are not proposed as mineralizers but show a very high IBP degradation, such as *Nocardia* sp. NRRL 5646 (1000 mg L^−1^ in 5d, [59]), *Variovorax* sp. Ibu-1 (500 mg L^−1^ in 7d, [60]), or the consortium *Comamonas aquatic* + *Bacillus* sp. (100 mg L^−1^ in 1.4d, [61]).

Some other strains show degradative efficiencies more similar to the nine bacteria under study using initial concentrations similar to those used in the present research (10 mg L^−1^), such as *Bacillus thuringiensis* B1 (2015b) [10,29], *Serratia marcescens* BL1 [11], *Citrobacter freundii* PYI-2 and *Citrobacter portucalensis* YPI-2 [62], *Pseudoalteromonas* sp. [63], *Rhizobium daejeonense* IBU_18 [33] or *Klebsiella pneumoniae* TIBU2.1, *Klebsiella variicola* c, *Pseudomonas aeruginosa* LOIBU1.2, and *Mycolicibacterium aubagnense* HPB1.1 [15].

Some others show lower IBP degradation efficiency compared to the nine bacteria under study: *Patulibacter* sp. I11 [31], *Pseudoxanthomonas* sp. DIN-3 [64], *Novosphingobium* sp. and *Pseudomonas* sp. [65], or *Nocardioides carbamazepine* sp. [30].

**Table 3:** Comparison of IBP degradation by the nine strains in this study and by other reported IBP-degrading bacteria.

Strain	Concentration (mg L^−1^)	Degraded Concentration (mg L^−1^)/Time (Days)	References
*Nocardia* sp. NRRL 5646	1000	1000/5 d	[59]
*Sphingomonas* sp. Ibu-2	500	500/3 d	[8,9]
*Patulibacter* sp. I11	1.0 0.25	0.28/12.5 d 0.125/12.5 d	[31]
*Variovorax* sp. Ibu-1	500	500/7 d	[60]
*Comamonas aquatic + Bacillus sp.*	100	100/1.38 d	[61]
*Bacillus thuringiensis* B1(2015b)	20	20/6 d	[10,29]
*Serratia marcescens* BL1	30	28/5 d	[11]
*Pseudoxanthomonas* sp. DIN-3	0.05	0.02/14 d	[64]
*Microccocus yunnanensis*	0.2	0.18/0.5 d	[12,34]
*Novosphingobium* sp. *Pseudomonas* sp.	0.062 0.082	0.062/3 d 0.082/8 d	[65]
*Rhodococcus cerastii* IEGM 1278	0.1 100	0.1/1.25 d 100/6 d	[35]
*Sphingopyxis granuli* RW412	456.5	456.5/3.1 d	[16]
*Citrobacter freundii* PYI-2 *Citrobacter portucalensis* YPI-2	8.0 8.0	8.0/15 d 8.0/15 d	[62]
*Bacillus siamensis* DSI-1	20	20/1 d	[66]
*Nocardioides carbamazepine* sp. Nov.	1.5	1.05/49 d	[30]
*Pseudoalteromonas* sp.	1.0	0.89/3 d	[63]
*Streptomyces murinus* D218 *Pseudomonas alloputida* M20	20 20	15.34/0.5 d 16.66/0.5 d	[67]
*Rhizobium daejeonense* IBU_18	1.5	1.36/28 d	[33]
*Microbacterium paraoxydans*	15	15/5 d	[23]
*Klebsiella pneumoniae* TIBU2.1	5	5/14 d	[15]
*Klebsiella variicola* LOIBU1.1	5	3/14 d	[15]
*Pseudomonas aeruginosa* LOIBU1.2	5	3.3/14 d	[15]
*Mycolicibacterium aubagnense* HPB1.1	5	2.9/14 d	[15]
*Labrys neptuniae* CSW11	1.0 5.0 10 100	1.0/4 d 5.0/4 d 10/7 d 48.4/28 d	[17]
*M. paraoxydans* CSW08	10	6.38/14 d	This study
*A. denitrificans* CSW15	10	4.69/7 d	This study
*P. citronellolis* CSW09	10	3.61/7 d	This study
*C. flacumfaciens* CSW18	10	3.22/28 d	This study
*B. tritici* CSW06	10	2.72/7 d	This study
*B. petrii* CSW07	10	2.45/28 d	This study
*S. zoogloeoides* CSW12	10	2.40/21 d	This study
*P. nitroreducens* CSW13	10	1.98/14 d	This study
*S. acidaminiphila* CSW10	10	0.9/28 d	This study

### 3.3. Phylogenetic Analysis of Ibuprofen-Degrading Bacterial Strains Based on 16S rRNA Gene

**Figure 3:**
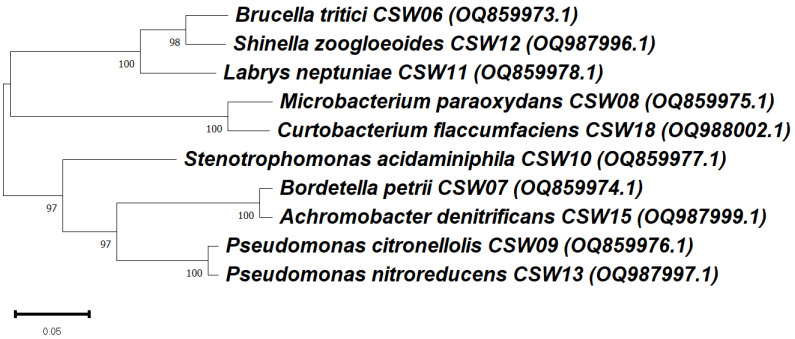
Maximum likelihood phylogenetic tree based on 16S rRNA gene sequences, showing the relationships among the different isolated bacterial strains under study. *Labrys neptuniae* CSW11 has also been included, as it was isolated from the same ibuprofen-degrading consortium C7. Bootstrap values at branch nodes indicate support based on 1000 replicates. The scale bar represents 0.05 substitutions per nucleotide. GenBank accession numbers are provided in parentheses.

A phylogenetic analysis was conducted to evaluate the evolutionary relationships among the nine bacterial strains under study, isolated from the IBP-degrading consortium C7 (Figure 3). The strain *Labrys neptuniae* CSW11, which was previously studied by Aguilar-Romero et al. [18], was included because it was also isolated from the same consortium C7.

The phylogenetic tree, constructed based on 16S rRNA gene sequences, revealed several well-supported clades, with bootstrap values ≥ 97%. Despite these close genetic relationships, significant variability was observed in the IBP degradation profiles among the isolates.

Although *L. neptuniae* CSW11, *B. tritici* CSW06 and *S. zoogloeoides* CSW12 are phylogenetic neighbors, their degradation efficiencies were quite different, since CSW11 exhibited a high degradation capacity, reaching 100% IBP removal (10 mg L^−1^) in only 7 days [18], while CSW6 and CSW12 showed lower degradation efficiencies, reaching only 27.2% and 24.0% IBP removal, respectively, after 28 days (Table 2). Similarly, although *M. paraoxydans* CSW08 and *C. flaccumfaciens* CSW18 are closely related, their degradation capacities differed substantially (63.8% vs. 32.2%, respectively). Strains belonging to *Pseudomonas* spp. (CSW09 and CSW13) also formed a distinct clade but exhibited differing degradation efficiency (36.1%, 19.8%).

These findings suggest that IBP degradation capacity is not strictly correlated with phylogenetic proximity. Instead, the ability to degrade IBP may depend on specific catabolic pathways or genes that are not universally distributed among closely related taxa. This highlights the potential role of horizontal gene transfer and ecological adaptation in shaping the functional capabilities of environmental bacteria involved in pharmaceutical bioremediation, although no genomic analysis was performed in this study.

In order to observe the taxonomic variety of the different bacterial strains that have been found to be IBP-degraders in the literature, Table 4 was constructed, which also includes the nine IBP-degrader strains described in the present study.

Seven of the nine IBP-degrader bacteria are included in the Phylum *Pseudomonadota*, with the majority of strains shown to mineralize IBP belonging to the family *Sphingomonadaceae* [8,9,13,14,16,68]. However, Wittich et al. [24] reported for the first time some *Pseudomonas* strains (*Pseudomonas citronellolis* RW422, RW423 and RW424, *Pseudomonadota/Gammaproteobacteria/Pseudomonadales/Pseudomonadaceae*) with the capacity to mineralize IBP. These seven strains, which belong to the Phylum *Pseudomonadota,* are included in different families: *Rhizobiaceae* (*S. zoogloeoides CSW12*), the same family as *Rhizobium daejeonense* IBU_18 [33]; *Xanthomonadaceae* (*S. acidaminiphila* CSW10), the same family as *Pseudoxanthomonas* sp. DIN-3 [64]; *Pseudomonadaceae* (*P. citronellolis* CSW09 and *P. nitroreducens* CSW13), the same family as *P. citronellolis* RW422 + RW423+ RW424 [24]; *P. alloputida* M20 [67] and *P. aeruginosa* LOIBU1.2 [15]. Three of the nine strains under study belong to the Phylum *Pseudomonadota*, but to families to which no other IBP-degrading bacterium belongs: *Brucellaceae* (*B. tritici* CSW06), *Alcaligenaceae* (*B. petrii* CSW07 and *A. denitrificans* CSW15). The two other bacteria (*M. paraoxydans* CSW08 and *C. flaccumfaciens* CSW18) belong to the Phylum *Actinomycetota* and both are included in the family *Microbacteriaceae*, the same as *M. paraoxydans* used by Show et al. [23] as an IBP-degrader. As far as we know, the rest of the IBP-degrader bacterial strains reported in the literature belong to the phylum *Bacillota* (family *Bacillaceae*, [10,29,61,66]). Taking into account that it is estimated that around 1300 bacterial phyla exist, although only 89 are recognized on the Silva database [69] and 41 are formally accepted by the LPSN [70], it can be deduced that the bacterial strains shown to degrade IBP are, at present, representatives of a very limited number of phyla.

### 3.4. Detection of the Main Metabolites in IBP Biotransformation by Isolated Bacteria

**Figure 4:**
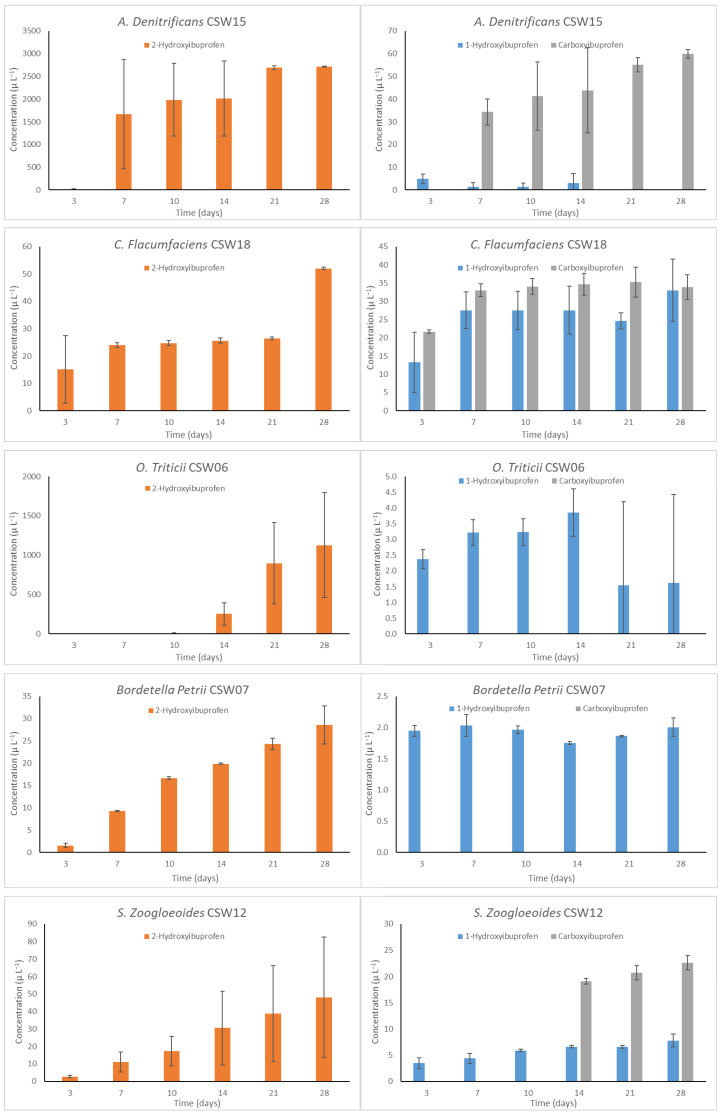

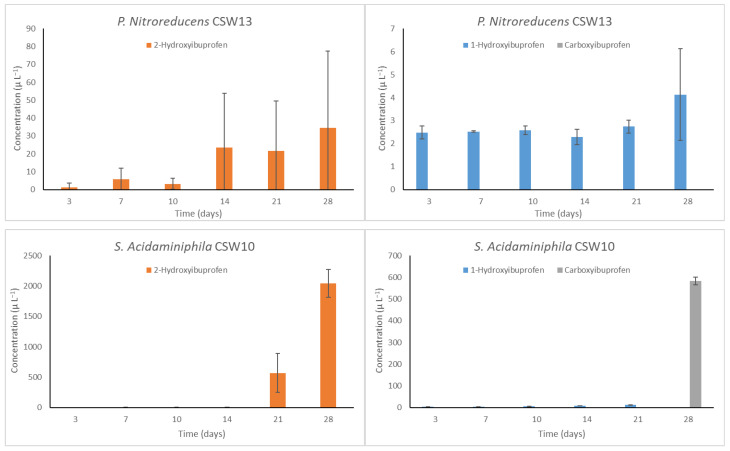
Ibuprofen biotransformation products detected during the degradation process. Error bars indicate standard deviation from three replicates.

IBP biotransformation products generated throughout the degradation process have been identified and are included in Figure 4 (the strains are shown in decreasing order of IBP degradation). Three metabolites were identified: 1-hydroxyibuprofen (1-OH- IBP), 2-hydroxyibuprofen (2-OH-IBP) and carboxyibuprofen (CBX-IBP). These are the same biotransformation products observed for IBP biodegradation by *L. neptuniae* CSW11, a bacterial strain also isolated from the C7 consortium [17].

Observing Figure 4, no general trend in the appearance and persistence of the three products in the system was observed, although the concentration of 2-OH-IBP was generally much higher than that of 1-OH-IBP. For some strains (CSW6, CSW10, CSW15), 2-OH-IBP concentrations reached up to 1000 µg L^−1^, while for others (CSW07, CSW12, CSW13, CSW15), values below 50 µg L^−1^ were observed, and these do not appear to be related to the concentration of IBP degraded.

In the case of 1-OH-IBP, values below 8 µg L^−1^ were obtained, except for CSW18, which reached up to 33 µg L^−1^. Both are products of IBP hydroxylation through the aliphatic side chains of the ring, with hydroxylation being the first reaction in IBP biodegradation. In general, 1- and 2-OH-IBP concentrations seem to increase throughout the degradation process, remaining in the system even for those strains with the highest degradation efficiency.

On the other hand, not all the isolates under study produced CBX-IBP, at least within the studied time range. Its occurrence does not appear to be related only to those bacteria that cause the most degradation. Several pathways for IBP degradation have been proposed for different bacterial strains, each one starting with one of these three metabolites [28,71]. But since two or three of these compounds are generally present [32,64], multiple degradation pathways may occur simultaneously.

Taking into account that in almost all of the degrading processes using the nine strains under study IBP degradation reached a plateau and did not continue to increase, this could indicate that the biotransformation products obtained remained in the system, which raises concerns about possible toxicity compared to that of IBP parent compound, although further ecotoxicology testing is required to confirm this. This toxicity was demonstrated in the case of *L. neptuniae* CSW11 [17], where the simultaneous presence of the same three biotransformation products was observed. However, Grabarczyk et al. [72] conducted an ecotoxicity screening evaluation of IBP and other selected pharmaceuticals and their transformation products on various organisms, observing that at least 2-OH-IBP and CBX-IBP are less toxic than IBP to *V. fischeri*, *L. minor, D. magna* and *R. subcapitata.* Specific conditions detailed by Salgado et al. [32] to detect a wide variety of other IBP metabolites and biotransformation products were also used, but no new metabolites were found in the present study.

## 4. Conclusions

Biodegradation of IBP using nine bacterial strains isolated from sewage sludge from a WWTP has been carried out, evaluating the degradation kinetics of each of them as well as the formation of the most relevant transformation products. All of them showed IBP degradative capacity in the presence of glucose as a secondary substrate, with an extent of degradation ranging from 9% to 64% in 28 days. The disappearance of IBP from water, along with the appearance of three biotransformation products (1-hydroxyibuprofen (1-OH-IBP), 2-hydroxyibuprofen (2-OH-IBP), and carboxyibuprofen (CBX-IBP)) throughout the process, confirms that these nine bacteria are IBP-degraders.

The tolerance of each strain to IBP present in the medium was quantified using the IC_50_ parameter, with values ranging between 38 and 1353 mg L^−1^, which are higher than those usually found even in WWTP waters, indicating a high level of IBP tolerance for all the strains studied. This is the first time that IC_50_ values are reported for IBP tolerance in the bacterial genera of the nine strains under study.

Phylogenetic analysis based on 16S rRNA gene sequences was conducted to explore the taxonomic affiliations of the nine bacteria under study in comparison to other bacterial strains previously found to be IBP-degraders. It was concluded that seven of the nine strains are included in the Phylum *Pseudomonadota*, the same as most of the IBP-degrader bacteria previously mentioned in the literature, but they belong to five different families: *Brucellaceae, Alcaligenaceae, Rhizobiaceae, Xanthomonadaceae,* and *Pseudomonadaceae*. The two other bacteria belong to the Phylum *Actinomycetota* and both are included in the family *Microbacteriaceae*.

Considering that the experiments were conducted in vitro, under controlled laboratory conditions and using small solution volumes, the results should be regarded as preliminary. At the laboratory scale, the method needs to be improved by incorporating consortia of other bacterial strains capable of degrading the toxic metabolites produced. This would lead not only to the degradation of IBP but also to its complete mineralization. Moreover, although the results suggest that these nine bacterial strains can degrade IBP in liquid media, they may not accurately reflect their behavior in more complex natural environments. On a larger scale or in situ, the bioremediation performance could differ significantly. Factors such as nutrient availability, pH, temperature, and the presence of other organisms and contaminants can all dramatically influence bacterial growth, motility, and interactions.

## Figures and Tables

**Table 1:** Inhibitory concentrations (IC_50_) of ibuprofen for specific degrading bacteria.

Bacteria	IC_50_ (mg/L)	R^2^
*B. tritici* CSW06	48	0.99
*B. petrii* CSW07	1353	0.98
*M. paraoxydans* CSW08	279	0.93
*P. citronellolis* CSW09	450	1
*S. acidaminiphila* CSW10	38	0.93
*S. zoogloeoides* CSW12	689	0.99
*P. nitroreducens* CSW13	1247	0.96
*A. denitrificans* CSW15	601	0.96
*C. flaccumfaciens* CSW18	908	0.94

**Table 2:** Kinetic parameters from ibuprofen biotransformation in solution 28 days after inoculation with different bacterial degrading strains and glucose (3 g L^−1^).

Bacterial Strain	Kinetic Model	K_1_ (d^−1^)	K_2_ (d^−1^)	tb (d)	DT_50_ (d)	Extent of Degradation (%)	R^2^	Err_scaled_	Calculated χ^2^ *
*M. paraoxydans* CSW08	HS	0.274	0.021	1.70	12.5	63.8	0.967	0.59	1.925
*A. denitrificans* CSW15	HS	0.434	0.002	1.34	44.7	46.9	0.993	0.05	0.225
*P. citronellolis* CSW09	HS	0.248	0.003	1.52	119	36.1	0.989	0.14	0.155
*C. flacumfaciens* CSW18	SFO	0.013	-	-	51.6	32.2	0.986	1.01	0.120
*B. tritici* CSW06	HS	0.226	0.000	1.40	426	27.2	0.987	0.29	0.113
*B. petrii* CSW07	SFO	0.011	-	-	61.2	24.5	0.857	3.08	1.029
*S. zoogloeoides* CSW12	HS	0.134	0.003	1.38	150	24.0	0.995	0.05	0.020
*P. nitroreducens* CSW13	HS	0.132	0.000	1.36	907	19.8	0.974	0.25	0.292
*S. acidaminiphila* CSW10	HS	0.083	0.001	0.83	669	9.02	0.805	0.37	0.143

* χ^2^ calculated values < χ^2^ corresponding tabulated value 12,592 (*p* < 0.05).

**Table 4:** Taxonomic classification of ibuprofen-degrading bacteria reported in the literature and those identified in this study.

Strain	Phylum/Class/Order/Family	Reference
*Sphingomonas* sp. Ibu-2	Pseudomonadota/Alphaproteobacteria/Sphingomonadales/Sphingomonadaceae	[8,9]
*Sphingobium yanoikuyae*	Pseudomonadota/Alphaproteobacteria/Sphingomonadales/Sphingomonadaceae	[13]
*Novosphingobium*	Pseudomonadota/Alphaproteobacteria/Sphingomonadales/Sphingomonadaceae	[65]
*Rhizorhabdus wittichii* MPO218	Pseudomonadota/Alphaproteobacteria/Sphingomonadales/Sphingomonadaceae	[14]
*Sphingopyxis granuli* TFA	Pseudomonadota/Alphaproteobacteria/Sphingomonadales/Sphingomonadaceae	[68]
*Sphingopyxis granuli* RW412	Pseudomonadota/Alphaproteobacteria/Sphingomonadales/Sphingomonadaceae	[16]
*Rhizobium daejeonense* IBU_18	Pseudomonadota/Alphaproteobacteria/Hyphomicrobiales/Rhizobiaceae	[33]
*Shinella zoogloeoides* CSW12	Pseudomonadota/Alphaproteobacteria/Hyphomicrobiales/Rhizobiaceae	This study
*Labrys neptuniae* CSW11	Pseudomonadota/Alphaproteobacteria/Hyphomicrobiales/Xanthobacteraceae	[17]
*Brucella tritici* CSW06	Pseudomonadota/Alphaproteobacteria/Rhizobiales/Brucellaceae	This study
*Variovorax* sp. Ibu-1	Pseudomonadota/Betaproteobacteria/Burkholderiales/Comamonadaceae	[60]
*Comamonas aquatic (+ Bacillus sp.)*	Pseudomonadota/Betaproteobacteria/Burkholderiales/Comamonadaceae	[61]
*Bordetella petrii* CSW07	Pseudomonadota/Betaproteobacteria/Burkholderiales/Alcaligenaceae	This study
*Achromobacter denitrificans* CSW15	Pseudomonadota/Betaproteobacteria/Burkholderiales/Alcaligenaceae	This study
*Serratia marcescens* BL1	Pseudomonadota/Gammaproteobacteria/Enterobacterales/Yersiniaceae	[11]
*Pseudoxanthomonas* sp. DIN-3	Pseudomonadota/Gammaproteobacteria/Xanthomonadales/Xanthomonadaceae	[64]
*Stenotrophomonas acidaminiphila CSW10*	Pseudomonadota/Gammaproteobacteria/Xanthomonadales/Xanthomonadaceae	This study
*Pseudoalteromonas* sp.	Pseudomonadota/Gammaproteobacteria/Alteromonadales/Pseudoalteromonadaceae	[63]
*Pseudomonas citronellolis* RW422 + RW423+ RW424	Pseudomonadota/Gammaproteobacteria/Pseudomonadales/Pseudomonadaceae	[24]
*Pseudomonas alloputida* M20	Pseudomonadota/Gammaproteobacteria/Pseudomonadales/Pseudomonadaceae	[67]
*Pseudomonas aeruginosa* LOIBU1.2	Pseudomonadota/Gammaproteobacteria/Pseudomonadales/Pseudomonadaceae	[15]
*Pseudomonas citronellolis* CSW09	Pseudomonadota/Gammaproteobacteria/Pseudomonadales/Pseudomonadaceae	This study
*Pseudomonas nitroreducens* CSW13	Pseudomonadota/Gammaproteobacteria/Pseudomonadales/Pseudomonadaceae	This study
*Citrobacter freundii* PYI-2	Pseudomonadota/Gammaproteobacteria/Enterobacterales/Enterobacteriaceae	[62]
*Citrobacter portucalensis* YPI-2	Pseudomonadota/Gammaproteobacteria/Enterobacterales/Enterobacteriaceae	[62]
*Klebsiella pneumoniae* TIBU2.1	Pseudomonadota/Gammaproteobacteria/Enterobacterales/Enterobacteriaceae	[15]
*Klebsiella variicola* c	Pseudomonadota/Gammaproteobacteria/Enterobacterales/Enterobacteriaceae	[15]
*Patulibacter* sp. I11	Actinomycetota/Thermoleophilia/Solirubrobacterales/Patulibacteraceae	[31]
*Microccocus yunnanensis*	Actinomycetota/Actinomycetes/Micrococcales/Micrococcaceae	[12,34]
*Patulibacter medicamentivorans*	Actinomycetota/Thermoleophilia/Solirubrobacterales/Patulibacteraceae	[32]
*Rhodococcus cerastii* IEGM 1278	Actinomycetota/Actinomycetia/Mycobacteriales/Nocardiaceae	[35]
*Nocardioides* carbamazepine sp.	Actinomycetota/Actinomycetia/Propionibacteriales/Nocardioidaceae	[30]
*Streptomyces murinus* D218	Actinomycetota/Streptomycetales/Streptomycineae/Streptomycetaceae	[67]
*Mycolicibacterium aubagnense* HPB1.1	Actinomycetota/Actinomycetes/Mycobacteriales/Mycobacteriaceae	[15]
*Nocardia* sp. NRRL 5646	Actinomycetota/Actinomycetes/Mycobacteriales/Nocardiaceae	[59]
*Microbacterium paraoxydans*	Actinomycetota/Actinomycetes/Micrococcales/Microbacteriaceae	[23]
*Microbacterium paraoxydans* CSW08	Actinomycetota/Actinomycetes/Micrococcales/Microbacteriaceae	This study
*Curtobacterium flaccumfaciens* CSW18	Actinomycetota/Actinomycetes/Micrococcales/Microbacteriaceae	This study
*Bacillus sp. (+Comamonas aquatic)*	Bacillota/Bacilli/Bacillales/Bacillaceae	[61]
*Bacillus thuringiensis* B1(2015b)	Bacillota/Bacilli/Bacillales/Bacillaceae	[10,29]
*Bacillus siamensis* DSI-1	Bacillota/Bacilli/Bacillales/Bacillaceae	[66]

## Data Availability

The original contributions presented in the study are included in the article/Supplementary Materials; further inquiries can be directed to the corresponding author.

## Supplementary Materials

The following supporting information can be downloaded at: https://www.mdpi.com/article/10.3390/microorganisms13081927/s1, Table S1. Optimized MRM parameters of the QqQ-MS determination of ibuprofen and its metabolites.
