# Supplementary material for: Removal of Ibuprofen from Contaminated Water by Bioaugmentation with Novel Bacterial Strains Isolated from Sewage Sludge
Source: Microorganisms. 2025 Aug 18;13(8):1927. doi: 10.3390/microorganisms13081927 (PMC12388369; doi:10.3390/microorganisms13081927)
Supplement: Supplementary file 1 [file microorganisms-13-01927-s001.zip › microorganisms-3776668-supplementary.pdf]

Table S1. Optimized MRM parameters of the QqQ-MS determination of ibuprofen and its metabolites

**Compound**	**Retention time (min)**	**Ion polarity**	**Precursor ion (m/z)**	**MRM 1 (quantification)**	**MRM 2 (confirmation)**	**CE (V)**
Ibuprofen	8.80	Negative	205	205>205	205>161	0/4
1-hydroxyibuprofen	7.84	Positive	240	240>205	240>163	8/20
2-hydroxyibuprofen	7.64	Positive	240	240>205	240>107	12/36
Carboxyibuprofen	7.63	Positive	254	254>219	254>117	8/48

CE: collision energy; MRM: Multiple Reaction Monitoring
